# Health benefits and risks of fermented foods—the PIMENTO initiative

**DOI:** 10.3389/fnut.2024.1458536

**Published:** 2024-09-03

**Authors:** Smilja Todorovic, Asli Akpinar, Ricardo Assunção, Cornelia Bär, Simona L. Bavaro, Muzeyyen Berkel Kasikci, Julieta Domínguez-Soberanes, Vittorio Capozzi, Paul D. Cotter, Eun-Hee Doo, Burcu Gündüz Ergün, Mustafa Guzel, Hayriye S. Harsa, Emre Hastaoglu, Christèle Humblot, Bahtir Hyseni, Muge I. Hosoglu, Aline Issa, Barçın Karakaş-Budak, Sibel Karakaya, Harun Kesenkas, Erhan Keyvan, Ibrahim E. Künili, Mary-Liis Kütt, Marta Laranjo, Sandrine Louis, Fani T. Mantzouridou, Antonia Matalas, Baltasar Mayo, Sandra Mojsova, Arghya Mukherjee, Anastasios Nikolaou, Fatih Ortakci, Diana Paveljšek, Giancarlo Perrone, Eugenia Pertziger, Dushica Santa, Taner Sar, Isabelle Savary-Auzeloux, Clarissa Schwab, Małgorzata Starowicz, Marko Stojanović, Michail Syrpas, Jyoti P. Tamang, Oktay Yerlikaya, Birsen Yilmaz, Jeadran Malagon-Rojas, Seppo Salminen, Juana Frias, Christophe Chassard, Guy Vergères

**Affiliations:** ^1^Institute for Biological Research Sinisa Stankovic, National Institute of Republic of Serbia, University of Belgrade, Belgrade, Serbia; ^2^Department of Food Engineering, Manisa Celal Bayar University Faculty of Engineering and Natural Science, Manisa, Türkiye; ^3^Egas Moniz Center for Interdisciplinary Research (CiiEM), Egas Moniz School of Health and Science, Almada, Portugal; ^4^Competence Division Method Development and Analytics, Agroscope, Berne, Switzerland; ^5^Institute of Sciences of Food Production (ISPA), National Research Council (CNR), Bari, Italy; ^6^STLO, INRAE, Institut Agro-Rennes Angers, Rennes, France; ^7^Facultad de Ingeniería, Universidad Panamericana, Aguascalientes, Mexico; ^8^Department of Food Science, University of Foggia, Foggia, Italy; ^9^Department of Food Biosciences, Teagasc Food Research Centre, Fermoy, Ireland; ^10^School of Living and Environmental Engineering, Dongyang Mirae University, Seoul, Republic of Korea; ^11^Biotechnology Research Center, Field Crops Central Research Institute, Ankara, Türkiye; ^12^Department of Food Engineering, Hitit University, Corum, Türkiye; ^13^Department of Food Engineering, Izmir Institute of Technology, Izmir, Türkiye; ^14^Cumhuriyet University, Sivas, Türkiye; ^15^French National Research Institute for Sustainable Development (IRD), Montpellier, France; ^16^Faculty of Food Technology, University “Isa Boletini”, Mitrovica, Republic of Kosovo; ^17^Biotechnology Institute, Gebze Technical University, Kocaeli, Türkiye; ^18^Faculty of Nursing and Health Sciences, Notre Dame University-Louaize, Zouk Mosbeh, Lebanon; ^19^Department of Food Engineering, Akdeniz University Faculty of Engineering, Antalya, Türkiye; ^20^Department of Food Engineering, Faculty of Engineering, Ege University, Izmir, Türkiye; ^21^Department of Dairy Technology, Faculty of Agriculture, Ege University, Izmir, Türkiye; ^22^Department of Food Hygiene and Technology, Faculty of Veterinary Medicine, Burdur Mehmet Akif Ersoy University, Burdur, Türkiye; ^23^Department of Fishing and Fish Processing Technology, Faculty of Marine Sciences and Technology, Canakkale Onsekiz Mart University, Canakkale, Türkiye; ^24^ÄIO, Tallinn, Estonia; ^25^MED-Mediterranean Institute for Agriculture, Environment and Development-CHANGE-Global Change and Sustainability Institute and Departamento de Medicina Veterinária-Escola de Ciências e Tecnologia (ECT), Universidade de Évora, Évora, Portugal; ^26^Department of Physiology and Biochemistry of Nutrition, Max Rubner-Institut, Karlsruhe, Germany; ^27^Laboratory of Food Chemistry and Technology, School of Chemistry, Aristotle University of Thessaloniki, Thessaloniki, Greece; ^28^Department of Nutrition and Dietetics, Harokopio University, Athens, Greece; ^29^Departamento de Microbiología y Bioquímica, Instituto de Productos Lácteos de Asturias (IPLA), Consejo Superior de Investigaciones Científicas (CSIC), Villaviciosa, Spain; ^30^Department of Food Safety and Veterinary Public Health, Food Institute, Faculty of Veterinary Medicine, Skopje, Ss. Cyril and Methodius University, Skopje, North Macedonia; ^31^Department of Molecular Biology and Genetics, Democritus University of Thrace, Alexandroupolis, Greece; ^32^Food Engineering Department, Istanbul Technical University, Istanbul, Türkiye; ^33^Institute of Dairy Science and Probiotics, Department of Animal Science, Biotechnical Faculty, University of Ljubljana, Domžale, Slovenia; ^34^Consiglio Nazionale delle Ricerche, Istituto di Scienze delle Produzioni Alimentari, Bari, Italy; ^35^Research Division Microbial Food Systems, Agroscope, Berne, Switzerland; ^36^Department of Epidemiology and Health Systems, Center for Primary Care and Public Health (Unisanté), University of Lausanne, Lausanne, Switzerland; ^37^Faculty of Agricultural Sciences and Food, Ss. Cyril and Methodius University in Skopje, Skopje, North Macedonia; ^38^Swedish Centre for Resource Recovery, University of Borås, Borås, Sweden; ^39^Human Nutrition Unit, INRAE, Université Clermont-Auvergne, Clermont-Ferrand, France; ^40^Department of Biological and Chemical Engineering, Aarhus University, Aarhus, Denmark; ^41^Department of Chemistry and Biodynamics of Food, Institute of Animal Reproduction and Food Research of the Polish Academy of Sciences, Olsztyn, Poland; ^42^Faculty of Medicine, University of Belgrade, Belgrade, Serbia; ^43^Department of Food Science and Technology, Kaunas University of Technology, Kaunas, Lithuania; ^44^Department of Microbiology, School of Life Sciences, Sikkim University, Gangtok, India; ^45^Department of Biological Sciences, Tata Institute of Fundamental Research, Hyderabad, India; ^46^Department of Nutrition and Dietetics, Faculty of Health Sciences, Çukurova University, Adana, Türkiye; ^47^Instituto Nacional de Salud de Colombia, Bogotá, Colombia; ^48^Functional foods Forum, Faculty of Medicine, University of Turku, Turku, Finland; ^49^Department of Technological Processes and Biotechnology, Institute of Food Science, Technology and Nutrition (ICTAN-CSIC), Madrid, Spain

**Keywords:** fermented foods, food characterization, food safety, functional claims, health benefits, health claims, mechanism of action, systematic review

## Abstract

Worldwide, fermented foods (FF) are recognized as healthy and safe. Despite the rapid increase of research papers, there is a lack of systematic evaluation of the health benefits and risks of FF. The COST Action CA20128 “Promoting innovation of fermented foods” (PIMENTO) aims to provide a comprehensive assessment on the available evidence by compiling a set of 16 reviews. Seven reviews will cover clinical and biological endpoints associated with major health indicators across several organ systems, including the cardiovascular, gastrointestinal, neurological, immune, and skeletal systems. Nine reviews will address broader biological questions associated with FF including bioactive compounds and vitamin production, nutrient bioavailability and bioaccessibility, the role of FF in healthy diets and personalized nutrition, food safety, regulatory practices, and finally, the health properties of novel and ethnic FF. For each outcome assessed in the reviews, an innovative approach will be adopted based on EFSA’s published guidance for health claim submissions. In particular, each review will be composed of three parts: (1) a systematic review of available human studies; (2) a non-systematic review of the mechanism of action related to the clinical endpoints measured by the human studies identified in part 1; and (3) a non-systematic review of the characterization of the FF investigated in the human studies identified in part 1. The evidence and research gaps derived from the reviews will be summarized and published in the form of a strategic road map that will pave the way for future research on FF.

## Introduction

### Need for coordinated research on FF—PIMENTO

The impact of food systems on planetary health is a societal issue of paramount importance requesting a transition towards more sustainable diets (1, 2). This transition resonates with the growing consumer demand for natural and healthier products available at affordable prices (3). On the other hand, while a body of evidence indicates the importance of dietary diversity for human health (4), biodiversity is decreasing worldwide (5). In times when diets are rapidly changing towards highly processed foods, there is a critical need to maintain and improve food as a source of diverse nutrients as well as beneficial microbes (6, 7).

FF hold a strategic place in all European diets due to the benefits they offer in terms of nutrition, cultural heritage, innovation, sustainability, and consumer interest. In particular, the potential of FF as part of the diet for improving human health has become highly relevant (8, 9). FF can meet the demands outlined above owing to their unique properties (10), which include the presence of live microorganisms and microbial metabolites, vitamins, and bioactive compounds as well as extended shelf-life. However, to fully realize their potential, there is a need for coordinating the wealth of new scientific evidence on FF emerging from the studies on food ecosystems, systemic approaches to food microbiology, foodomics, and nutrigenomics as well as on the role of the gut microbiota and the gastrointestinal (GI) tract in health and disease onset (9, 11). This consolidated knowledge and, therefore, FF can be ultimately integrated into effective public health and sustainability policies (12).

PIMENTO is a European initiative funded by the COST Action (CA20128) to address the needs indicated above through research on FF. In particular, PIMENTO contributes to an open-access identification, standardization, and mapping of existing and potential beneficial microorganisms and communities. PIMENTO also collectively advances the scientific evidence on the health effects of FF, building a risk–benefit approach and tightly connecting and clarifying the scientific evidence on the health-promoting properties of FF. Furthermore, PIMENTO contributes to the European Green Deal strategy “Farm to Fork” by enhancing research and innovation on fermentation-based solutions and processes, improving food’s nutritional, structural and functional properties. Through its multi-modal innovative approach, PIMENTO not only responds to the expectations of different European communities for healthy and sustainable food products by boosting the development of FF, but also places Europe as a leader in FF innovation worldwide.

## Innovative strategy to evaluate the health benefits and risks of FF

In line with PIMENTO’s mission Working Group 3 of PIMENTO (PIMENTO WG3) aims at evaluating the health benefits and risks of FF. Compared to other components of the human diet, FF add a unique dimension to the relationship between diet and health because this category of foods contains fermentative microorganisms and their fermentation products in addition to the macro- and micronutrients classically studied by nutritionists. Although nutrition science is an interdisciplinary field, published research reports are often characterized by a limited scope, resulting in a lack of holistic assessment of the complex array of elements that characterize the interaction of food components with the human body. This fragmented approach to nutrition research makes it difficult to objectively characterize the health properties of diets, foods, or nutrients. A pragmatic, yet innovative approach for the academic sector to tackle the health properties of diets is to use the “European Food Safety Authority” (EFSA) health claim guidelines (13, 14). The EFSA guidance requires not only that the results of human studies are systematically reported and evaluated but also an assessment of the properties of a food or ingredient for which the effect is claimed. This characterization comprises information on the composition and stability of the active compounds in the food of interest. Furthermore, EFSA asks for additional data on the mechanism of action to be provided, derived from *in vitro* or animal models. The mechanistic data should be complemented by information about the bioavailability and bioaccessibility of the active compounds.

Although fermented foods are diverse, underlying mechanisms contributing to their potential impact on health might be similar. A harmonized and systematic review on the scientific evidence for the health properties of FF can thus lead to the identification of common functional properties. PIMENTO WG3 will evaluate the health properties of FF by following an innovative approach that will be based on the compilation of a series of reviews based on the EFSA guidance for health claims. This strategy includes the three pillars human studies, product characteristics, and mechanism of action as well as two complementary topics closely associated with FF, i.e., food safety and the interaction between FF and gut microbiota.

In May 2022, a workshop was held in Belgrade to define the functional thematic areas that the PIMENTO COST Action should investigate. A series of 16 thematic areas covering the most relevant health indicators in key organ systems were selected. These thematic areas were organized into two groups according to whether they could be reviewed in close alignment with the EFSA guidance. Seven “EFSA projects” (E1-E7) were defined, covering the following clinical areas: GI health (E1), allergy (E2), immunity (E3), metabolic health (E4), cardiovascular health (E5), bone health (E6), and cognitive health (E7). Nine “Satellite projects” (S1-S9) were also formed, covering important topics on FF that are less amenable to adaptation to the EFSA guidance: bioactive compounds (S1), vitamin production (S2), nutrient bioavailability and bioaccessibility (S3), ethnic foods (S4), healthy diets (S5), personalized nutrition (S6), food safety (S7), novel foods (S8), and byproducts of fermentation (S9).

Following this initial phase, a first round of literature analyses was conducted to define a specific research question for each of the topics. These questions should be systematically addressed to assess the available evidence and identify knowledge gaps to focus future research. The final review questions are presented in Table 1. As the preliminary work progressed, the review process became increasingly harmonized and the distinction between the two categories of reviews blurred so that the 16 reviews were no longer referred to as EFSA or Satellite reviews. Finally, a generic study protocol (PIMENTO-SP; Supplementary File 1) was developed based on the PROSPERO guidance for systematic reviews (13, 15) and further adapted to integrate the non-systematic parts of the reviews. In addition, a generic search strategy of the literature in PubMed, Scopus, and the Cochrane Library Central (PIMENTO-LS) was developed to systematically search for human studies reporting on the health effects of FF (Supplementary File 2). PIMENTO-SP and PIMENTO-LS forms will be used as a framework for the content of each review and adapted according to the specifics of the biological or clinical endpoints being addressed.

**Table 1:** Overview of the thematic areas and review questions addressed by PIMENTO WG3.

Review number and thematic area	Research question
E1. Gastrointestinal health	Does consumption of fermented foods have a beneficial effect on gastrointestinal discomfort and normal defecation in a healthy adult population?
E2. Allergies	What is the effect of the consumption of fermented food on the development of food allergic symptoms in the food allergic population and a population at high risk for food allergy?
E3. Immunity	Can consumption of fermented foods aid in prevention of bacterial vaginosis and/or vulvovaginal candidiasis?
E4. Metabolic health	Does fermented foods consumption help to reduce postprandial glucose response or maintain fasting blood glucose concentrations or insulin sensitivity in healthy or prediabetic adults?
E5. Cardiovascular health	Does consumption of fermented dairy products impact blood lipids in healthy adults?
E6. Bone health	What is the effect of consuming fermented foods on the bone health of healthy adults and adults with osteoporosis and osteopenia?
E7. Cognitive health	Does the consumption of foods fermented with Lactobacillus sp. and/or Bifidobacterium sp. have a beneficial effect on cognitive performance in a healthy adult population, including adults with mild cognitive impairment?
S1. Bioactive compounds	What compounds derived from food fermentation are associated with effects on clinical endpoints in human studies?
S2. Production of vitamins	Does the consumption of fermented foods, fortified in vitamin by fermentation, contributes to vitamin coverage of a healthy population or of a vitamin-deficient population?
S3. Bioavailability and bioaccessibility of nutrients	Does sourdough and regular bread fermentation increase iron bioavailability, absorption, and status in humans?
S4. Ethnic foods	What are the health effects of ethnic fermented foods?
S5. Healthy diets	What is the impact of fermented foods consumption on mortality risk?
S6. Personalized nutrition	Does the impact of fermented foods on different health outcomes depend on specific characteristics of population groups, and, consequently, can fermented foods be used in tailored nutritional strategies?
S7. Food safety	What are the main microbiological and chemical hazards posed by fermented foods and their associated risks?
S8. Novel fermented foods	What are the health effect of novel fermented foods?
S9. Food byproducts	What are the health effect of fermented whey?

E1–E7 refer to each of the seven “EFSA projects.” S1–S9 refer to each of the nine “Satellite projects.”

A summary of the aims and methodology of each of the 16 reviews under preparation is presented in the following sections. Although the sections below focus their research summary on the analysis of the human clinical studies, each of the 16 projects will also address the non-systematic parts discussed above. Specific study protocols were prepared for each review and were deposited in the Open Science Framework (OSF). In addition to the review plans, the study protocols document the names and institutes of the reviewing teams.

## Research questions addressed by the PIMENTO WG3 reviews

### Gastrointestinal health

In recent years, FF have been the focus of a resurgence in interest among the general public, primarily owing to their purported health benefits. Indeed, growing evidence shows that FF positively modulates GI health through diverse mechanisms, including the potential probiotic effect of their constituent microbes, reduction of anti-nutrients, and bioactivity of novel compounds produced during fermentation, among others (16). Concerning the former, it is notable that several probiotic microbes, which are often closely related to those found in FF (17, 18), have been shown to be beneficial in the management of GI symptoms in different populations, such as pregnant women, athletes, and patients with irritable bowel syndrome (IBS) and neurodegenerative disorders (19–22). However, despite this growing body of evidence, a systematic review of the impact of FF consumption on GI discomfort and GI functions, such as defecation, in healthy adults has not been conducted.

This systematic review will address the following question: *“Does consumption of fermented foods have a beneficial effect on gastrointestinal discomfort and normal defecation in a healthy adult population?”* To answer this research question, we will investigate the effect of FF consumption on GI discomfort and defecation as guided by the “Guidance on the scientific requirements for health claims related to the immune system, the gastrointestinal tract and defense against pathogenic microorganisms” (23). According to EFSA, symptoms associated with GI discomfort include abdominal pain, cramps, bloating, straining, borborygmi (rumbling) and a sensation of incomplete evacuation, along with excessive intestinal gas accumulation. Reducing GI discomfort is considered an indicator of improved GI function and, hence, a beneficial physiological effect (23). Similarly, maintenance of normal defecation is regarded as a beneficial physiological effect for the general population, given that it does not result in diarrhea. Therefore, the review will additionally investigate the effect of FF consumption on defecation in the context of maintenance or facilitation of normal defecation through one or more of the following means: improved frequency of bowel movements, increased fecal bulk, improving stool consistency, or decreasing transit time.

OSF link to study protocol: PIMENTO-SP-E1.

### Allergies

Food allergy (FA) is an abnormal reaction of the immune system after exposure to specific foods, which is a serious food safety and public health problem worldwide. Currently, food allergies affect up to 10% of children and 2–3% of adults and appear to be increasing in prevalence across the world (24). Based on the immunological mechanisms involved, FA may be classified as (1) IgE-mediated, the most well-understood form, which is caused by IgE antibodies against food antigens; (2) non-IgE-mediated, in which the immune response is thought to act mainly through cell-mediated mechanisms; (3) or mixed, in which both IgE-mediated and cell-mediated immunological mechanisms are involved (25). The IgE-mediated allergies occur most frequently in the first years of life and may lead to urticaria, angioedema, oral allergic syndrome, rhinitis, or acute asthma and anaphylaxis, while cutaneous, respiratory, or GI reactions characterize non-IgE allergies.

The mechanism of action of FA usually occurs in two different phases: a sensitization phase and an elicitation phase, with the latter resulting in clinical symptoms of allergic disease (26). Sensitization is the phase during which immunological priming to the inducing allergen occurs, associated with the evolution of a Th2-biased immune response. If the sensitized subject is subsequently exposed to a sufficient amount of the inducing food allergen, an allergic reaction may be elicited, resulting in the clinical manifestations described above.

Food processing can impact the IgE-binding properties of food proteins’ antigenic sites and their ability to elicit allergic reactions (27). Compared to other food processing techniques, food fermentation has shown unique advantages in reducing the allergenicity of foods; thus, it may represent a new trend in preventing food-induced allergies in child and adult populations. For example, consuming fully matured Parmigiano Reggiano cheese seems to inhibit the IgE binding to milk allergens and be well tolerated by a subset of cow’s milk allergic patients (28). In the same way, the consumption of fermented dairy products appears to be associated with reduced asthma and atopic dermatitis in children born by caesarean section and affected by FA (29). Similarly, wheat-based sweet baked goods with gluten degraded by sourdough fermentation seem to be well tolerated by patients affected by coeliac disease (30).

Although the consumption of FF might be an effective way to reduce the severity of allergic reactions, a systematic review is needed to evaluate the evidence more objectively. This review will thus address the following question: *“What is the effect of the consumption of fermented food on the development of food allergic symptoms in the food allergic population and a population at high risk for food allergy?”*

OSF link to study protocol: PIMENTO-SP-E2.

### Immunity

Human clinical studies on immunity investigate a broad array of topics, some being addressed in other PIMENTO WG3 reviews (e.g., allergy, gut health). With regard to pathogenic diseases, immunity refers to the safeguarding of health through the prevention of dysbiosis. This highlights the importance of maintaining a balance of commensal microorganisms to sustain immune function.

As true for other parts of the human body, the composition of the community of microbial residents influences a balanced immune response in the female genital system. Bacterial vaginosis (BV) and vulvovaginal candidiasis (VVC) are the most common pathogenic conditions in women that not only cause discomfort but also result in increased susceptibility to other infections or risk of obstetric complications (31). Conventional therapies for BV and VVC involve oral and topical application of antimicrobial agents with a high risk of recurrence (32). Both conditions arise when the natural microbiota is disturbed. Increasing the proportion of lactobacilli and decreasing the proportion of potentially pathogenic bacteria or yeasts in the vagina thus becomes the target for prevention and treatment of both conditions.

Clinical studies have demonstrated the efficacy of oral administration of specific probiotics (33), yet the mechanism of translocation remains to be elucidated. A few human studies suggest the beneficial effects of the consumption of FF for the prevention and treatment of vaginitis. However, clinical sciences and food sciences take a distinct approach to using bacteria, with the former focusing on efficacy studies involving probiotic strains as supplements and the latter employing the same strains as culture organisms to ferment food matrices. This systematic review aims to address this gap and focus on the potential role of FF in the diet to modulate vaginitis by answering the question: “*Can consumption of fermented foods prevent bacterial vaginosis or vulvovaginal candidiasis?”*

OSF link to study protocol: PIMENTO-SP-E3.

### Metabolic health

Type 2 diabetes (T2D) is one of the major diseases among metabolic disorders. The prevalence of T2D is rising worldwide and represents a significant burden for the healthcare system (34). The World Health Organization (WHO) defines diabetes as a condition with elevated fasting plasma glucose and elevated postprandial plasma glucose in the oral glucose tolerance test (35). T2D is often characterized by insulin resistance and hyperinsulinemia, which contributes to the disease development.

Several studies review the effect of specific FF, particularly fermented milk, on glycaemia parameters, among other health outcomes. Other reviews consider several kinds of fermented foods but include different studies independently of the health status of the participants (36–38). In this context, a systematic review of the effects of consumption of different FF on glucose homeostasis in a healthy population is, to our knowledge, missing. By conducting a systematic search of human studies, this systematic review aims to assess whether regular FF consumption positively impacts T2D metabolic risk factors in healthy adults without medication, including participants with prediabetes and obesity. The main outcomes will include glucose and insulin homeostasis parameters, such as the maintenance of normal fasting blood glucose levels or a postprandial glucose response. The review will address the following question: *“Does fermented foods consumption help to reduce postprandial glucose response or maintain fasting blood glucose concentrations or insulin sensitivity in healthy or prediabetic adults?”*

OSF link to study protocol: PIMENTO-SP-E4.

### Cardiovascular health

Cardiovascular diseases (CVD) remain the leading cause of morbidity and mortality worldwide, with significant contribution to loss of health and excessive healthcare costs (39). Noticeably, deaths from CVD nearly doubled from 12.1 million in 1990 to 20.5 million in 2021, accounting for 32% of all deaths. Over three-quarters of CVD deaths take place in low- and middle-income countries (40). Diet has a significant impact both on disease pathogenesis and prevention. Although several cohort studies have examined the association of objective endpoints (e.g., CVD incidence or mortality) with fermented dairy foods intake, the results remained unclear, as suggested by null or inverse associations (38, 41).

Specific blood lipids, such as lipoproteins and triglycerides, are risk factors for CVD (42). Although fermented dairy products are an important part of the diet worldwide, the impact of their consumption on blood lipid profile in healthy adults is unclear. This review aims to address whether fermented dairy product consumption can impact the blood lipid profile in healthy adults. Among all fermented food groups, fermented dairy products may provide the best scientific evidence for a positive impact of fermented foods on cardiovascular health through their action on circulatory lipids. In addition, fermented dairy products are relatively well characterized and bioactive compounds have been identified that may mediate their impact on cardiometabolic health (43). The focus on fermented dairy products is thus motivated by their potential to provide key elements in this review, which will cover all three elements of the reviewing strategy, namely human studies, product characteristics, and mechanism of action. All human studies with adults without medication and disease will be considered. The primary outcome investigated will be the blood lipid profile, which includes total cholesterol, HDL-c, LDL-c, triglycerides, or their combination. Secondary outcomes will include blood glucose and insulin levels, hs-CRP, TNF-alpha, IL-1, IL-6, blood pressure, and anthropometric measurements, including body mass index (BMI). The review will address the following question: *“Does consumption of fermented dairy products impact blood lipids in healthy adults?”*

OSF link to study protocol: PIMENTO-SP-E5.

### Bone health

Bones undergo continuous remodeling and repair, and their proper function relies on the intake of essential dietary nutrients, with calcium being of paramount importance (44). Calcium consumption alone is insufficient to maintain bone health unless it is efficiently absorbed. Many factors, such as age, health status, or pregnancy, may influence absorption. Further, dietary calcium is absorbed in the small intestine in its ionized form (Ca^2+^). This process depends on the intestinal pH, a factor modulated by diet as the pH of the chyme rapidly rises during the digestion process as it enters the small intestine, potentially hindering the absorption of ions (45).

FF have garnered substantial attention as potential dietary interventions against osteopenia and osteoporosis (46, 47). As of milk origin, dairy products are excellent sources of calcium (48). The low pH found in most dairy FF could delay the neutralization of chyme, thus prolonging calcium ionization and enhancing its absorption. In plant-based foods, the absorption of calcium (and other minerals) can be adversely affected by anti-nutritional factors such as oxalates and phytates, whose levels can be reduced by fermentation (49). Some of the metabolites resulting from the fermentation processes also possess the potential to modulate the physiology of cells, including bone cells. Association studies have highlighted the significant role of vitamins in maintaining bone health, including vitamin D, vitamin K, and B-group vitamins (50). FF stands out as a potentially rich source of both vitamin K (menaquinones) and B-group vitamins (such as riboflavin and folate). These vitamins are produced by fermentative microorganisms, particularly those in the lactic acid bacteria (LAB) group. Additionally, FF could modulate the gut microbiota, emerging as a factor in bone homeostasis and the pathogenesis of osteoporosis (51).

In light of these insights, a systematic review will be conducted to comprehensively evaluate the existing scientific evidence on the effect of FF consumption on bone health. The evidence will be drawn from randomized clinical trials (RCT) and observational studies involving healthy adults and adults diagnosed with either osteoporosis or osteopenia. The central research question guiding this review is: *“What is the effect of consuming fermented foods on the bone health of healthy adults and adults with osteoporosis and osteopenia?”*

OSF link to study protocol: PIMENTO-SP-E6.

### Cognitive health

In today’s performance-oriented world, where lifespan is extending and, as a result, the health burden on the older population is increasing, maintaining cognitive health throughout life into old age is critical. In recent years, research on the microbiota-gut-brain axis has provided insights into the effects of gut microbiota on cognitive health. As a result, the effects of consuming FF containing beneficial microbes has become an active area of research.

Consumption of FF may affect the central nervous system function, although the mechanisms underlying the effect of FF on cognitive performance are still unknown. Three non-exclusive hypotheses have been proposed regarding the beneficial effects of fermentation on brain health, each relying on data derived primarily from probiotic studies (52). First, fermentation modulates the production and bioavailability of compounds such as bioactive peptides and phytochemicals (53). Among these, short-chain fatty acids (SCFA) positively influence the host’s metabolism through their effect on the central nervous and the immune systems (54). The second hypothesis is focused on the neurobiology of the stress response through the association of the hypothalamic–pituitary–adrenal (HPA) axis and the gut microbiota: probiotic administration appears to improve cognitive function by normalizing the HPA activity and reducing systemic inflammation (55). The third hypothesis suggests that FF may control the release of neurotransmitters and thus affect cognitive performance. Taken together, the ability of bacteria to alter the chemical composition of foods and, upon consumption, the composition of the gut microbiota community may account for the beneficial effects of FF on human cognitive performance.

In this context, the probiotic properties of *Bifidobacterium* sp. and *Lactobacillus* sp. have recently attracted attention, as several strains have been reported to have beneficial effects on cognitive abilities (52). Bioactive compounds synthesized by these bacteria that may directly or indirectly affect cognitive performance, include neurotransmitters (e.g., gamma-aminobutyric acid (GABA), serotonin), SCFA (e.g., butyrate, acetate), peptides, and vitamins (56). As these bacterial species are commonly present in FF, a systematic literature review will be conducted to evaluate the cognitive performance of humans who consume these food products. This review will thus address the question: “*Does the consumption of foods fermented with Lactobacillus* sp. and/or *Bifidobacterium* sp. *have a beneficial effect on cognitive performance in a healthy adult population, including adults with mild cognitive impairment?”*

OSF link to study protocol: PIMENTO-SP-E7.

### Bioactive compounds

Bioactive compounds, such as polyphenols, peptides, exopolysaccharides, conjugated linoleic acid (CLA), or vitamins, are present in foods across all food groups and cover a broad range of biological activities, such as antioxidant, anti-inflammatory, antimicrobial, and antihypertensive effects (57). Food fermentation offers a unique opportunity to increase the concentration of bioactive compounds in foods (58, 59). Many publications highlight the role of fermentation in producing bioactive molecules, potentially explaining the beneficial effects of FF intake on different aspects of health (60–63). However, evidence linking these bioactive compounds in FF to their effect on clinical endpoints in human studies is scarce and has not been systematically addressed.

This systematic narrative review aims at cataloguing compounds in FF for which evidence of bioactivity has been reported in human studies by addressing the following research question: “*What compounds derived from food fermentation are associated with effects on clinical endpoints in human studies?”* This question will be answered by searching all human studies, specifically investigating the potential role of one or several bioactive compounds present in FF on clinical outcomes. A catalogue of the FF, for which an association between the bioactive compounds and clinical endpoints has been reported, will be established.

OSF link to study protocol: PIMENTO-SP-S1.

### Production of vitamins

Vitamins are essential micronutrients required for many vital human functions. The human body cannot synthesize most vitamins due to its limited biosynthetic capacity and requires external provision through the diet. Despite the presence of vitamins in a wide range of foods, deficiencies remain high in many contexts, leading to health problems specific to each vitamin (64). For example, vitamin A deficiency results in visual impairment, frailty, and increased mortality risk, while vitamin B9 (folate) can lead to neural tube defects in the offspring. Conventional treatment and prevention approaches, such as supplementation and fortification, have not always proven effective and may have undesirable side effects (64, 65).

FF have the potential to provide a range of health benefits, including vitamin synthesis (66). The scientific literature also reports on the possibility of increasing vitamin levels in the general population by consuming FF naturally enriched in vitamins as an alternative dietary approach to fortification or supplementation (67, 68). Thus, this systematic narrative review aims to investigate if consuming foods enriched in vitamins through fermentation contributes to the vitamin needs of a healthy population with varying vitamin status. The specific research question will be: *“Does the consumption of fermented foods, fortified in vitamin by fermentation, contributes to vitamin coverage of a healthy population or of a vitamin-deficient population?”* In the human studies included in the review, the primary outcome of interest will be the vitamin status in the study participants assessed in relation to FF consumption, while the secondary outcome will be the vitamin content in FF.

OSF link to study protocol: PIMENTO-SP-S2.

### Bioavailability and bioaccessibility of nutrients

Micronutrient deficiencies are an alarming global health problem. In particular, iron deficiency anemia affects a large part of the world’s population, mainly children and pregnant women, and is considered one of the leading contributors to the global disease burden (69). Since there is no physiological mechanism for excretion, the iron balance in the human body is only controlled by absorption (70). Phytate, polyphenols, calcium, ascorbic acid, and muscle tissue are dietary factors that have been repeatedly shown to affect iron absorption in several studies (71, 72).

Evidence suggests that fermentation significantly improves the bioavailability of iron in foods, and especially of plant-based foods such as cereals and legumes, which often serve as important sources of iron in many diets (73). Sourdough and yeast-leavened breads are staple foods produced in many parts of the world. Fermentation can enhance the bioavailability of non-hem iron in sourdough bread (74) through various mechanisms, such as reducing phytate levels, increasing acidity, and generating organic acids.

The primary purpose of this systematic review is to comprehensively examine the available clinical studies that assessed the association between fermented sourdough and yeast-leavened bread and iron bioavailability, absorption, and status in human subjects. This review will address the following question: *“Does sourdough and regular bread fermentation increase iron bioavailability, absorption, and status in humans?”* The search strategy and inclusion criteria will be determined *a priori*. More specifically, all types of sourdough and yeast-leavened bread, prepared from cereal flour(s) that may or may not have been previously fortified will be considered exposure or intervention. The outcomes of interest will include iron-related biomarkers (i.e., hemoglobin, hematocrit, serum iron, serum ferritin, serum transferrin receptor, total iron-binding capacity, or iron isotope absorption). Overall, the review is anticipated to contribute to a better understanding of the impact of fermentation on iron bioavailability in these staple food products.

OSF link to study protocol: PIMENTO-SP-S3.

### Ethnic fermented foods

According to the United Nations, the world population may increase to 9.7 billion in the next five decades (75). The already existing problem of inadequate access to nutritious food for everyone will be further exacerbated. It is also projected that in 2030, over 670 million people will be experiencing hunger. Finding alternative solutions to ensure a healthy food environment for everyone is thus of paramount importance.

Ethnic FF can be defined as foods originating from the heritage and culture of an ethnic group of people who use their ethno-microbiological knowledge of food fermentation (76) with local ingredients from plant or animal sources (77). Ethnic FF are often artisanal and thus at risk of disappearing together with the diversity of native microorganisms used in their production due to urbanization and changing food habits (76).

The scientific literature suggests that consuming ethnic FF like koumiss, kimchi, and doenjang may positively affect health, including improvements in hematological and biochemical factors. Ethnic FF may reduce triglycerides and cholesterol levels, increase high-density lipoprotein (HDL) cholesterol, improve blood glucose control and, consequently, improve health and well-being (78–81). While specific ethnic FF like natto, kinema, kimchi, tempeh, and pulque have received substantial attention in research, many other ethnic FF are neglected despite their potential health benefits.

Regardless of the wide variety of ethnic FF produced and consumed worldwide (76, 82), a comprehensive review examining the health benefits and potential risks associated with these foods has yet to be conducted. This narrative systematic review will thus address the following question: *“What are the health effects of ethnic fermented foods?”* In answering the review question, a list of ethnic FF based on published literature will be first compiled (76, 82), followed by identifying clinical studies associating the consumption of ethnic FF with clinical endpoints irrespective of the health condition studied. Additional information on the characteristics of the ethnic FF, including their region of origin, will also be presented.

OSF link to study protocol: PIMENTO-SP-S4.

### Healthy diets

Diet composition is one of the most important determinants of mortality risk (83). Fermented dairy products and other FF are currently being studied for their potential health effects in numerous clinical studies, evaluating the effects of FF on chronic disease risk factors, immune system performance, and cognitive function. However, only a few studies have focused on the relationship between FF consumption and life expectancy or mortality (17). Therefore, this review aims to evaluate the evidence on the association between the consumption of FF and mortality risk in free-living populations and answer the question: *“What is the impact of fermented foods consumption on mortality risk?”*

A systematic review of the literature, incorporating a meta-analysis if a significant number of pertinent human studies are available, will be undertaken. The evaluation will focus on cohort studies with the primary objective of uncovering the impact of diets, including FF, on mortality. Mortality rate, mortality risk, and death rate will be extracted as primary outcomes of this review. Confounding factors such as demographic data, life quality indices, general health status, diet quality, alcoholic drink consumption and smoking habits, as well as chronic morbidity developed during the reported study, will be considered. Supporting evidence will be derived from studies that examined the beneficial health effects of fermented foods and the probable underlying mechanisms of action, with potential adverse effects also being noted (84).

The findings of this study will inform future research on the role of FF in healthy diets. Finally, it is expected to highlight the need for more detailed inclusion of FF in national and European dietary guidelines and population nutrition surveys.

OSF link to study protocol: PIMENTO-SP-S5.

### Personalized nutrition

The gut microbiota has recently been identified as a person-specific factor determining variable metabolic response to dietary intake (85). Interestingly, the fermentation of food by technological microorganisms and the transformation of ingested nutrients by the gut microbiota share common enzymatic reactions (86). Taken together, these analogies suggest that dietary fermented foods could also modulate the inter-individual variability in response to food intake (87, 88). This systematic narrative review aims to determine whether health outcomes in response to FF show a significant inter-personal variability. If health outcomes vary between individuals, FF can be considered as potential levers in personalized or precision nutrition strategies. According to the recent Food Forum of the National Academies of Sciences, Engineering, and Medicine (89), human variability in response to food can be regarded as a basis for developing personalized nutrition and targeted nutritional guidelines.

To this end, human studies (all populations studied) that include short- to long-term nutritional or dietary evaluations will be collected systematically. The review will thus focus on studies reporting variable health responses following the consumption of FF, which could be attributed to well-defined or yet-to-be-defined characteristics of populations. Additionally, considering that the field is relatively novel and exploratory, reviews or other types of publications (e.g., reports, position papers) in which dietary FF are discussed in relation to personalized or precision nutrition will also be included. Particular emphasis will be given to factors that may explain a variable health response: (1) populations (e.g., gender, age, location, physical activity), (2) health status (global or specific markers, with particular focus on gut microbiota composition or function), (3) evaluation of the diets and supplementations, including the detailed description of FF (e.g., type, nutritional composition, matrix description). This work will be a first step towards evaluating the feasibility of using FF in tailored nutritional strategies. This review will address the following question: *“Does the impact of fermented foods on different health outcomes depend on specific characteristics of population groups, and, consequently, can fermented foods be used in tailored nutritional strategies?”*

OSF link to study protocol: PIMENTO-SP-S6.

### Food safety

Foodborne diseases are illnesses that result from consuming contaminated food. Clinical signs and symptoms differ depending on the cause or hazard involved (90). Food safety is a high-priority topic, considering that sufficient amounts of safe, sustainably-produced food must be secured for a growing world population to comply with Sustainable Development Goal 12 of the 2030 Agenda of the United Nations (91). Fermentation is one of the oldest traditional processes for achieving safe food production (92); however, renewed interest in FF requires re-examining their safety. This systematic narrative review will ask the question: “*What are the main microbiological and chemical hazards posed by fermented foods and their associated risks?”* In other words, are fermented foods safe for humans?

A systematic review of the scientific literature will be performed to address this question. Reports associating the occurrence of food hazards of microbiological and/or chemical origin with the consumption of FF by the general population, including vulnerable groups (such as, pregnant and breastfeeding women, children, and elderly), will be compiled without limitation by age or gender. In addition to presenting the illnesses associated with FF consumption, the review will provide scientific evidence on 1) the occurrence (levels/magnitude, frequency) of hazards associated with the consumption of fermented foods; 2) the associated risks of exposure, taking into account their probability and severity; and 3) the reported outbreaks as well as the extent to which the diseases occur (93).

OSF link to study protocol: PIMENTO-SP-S7.

### Novel foods

Fermentation confers functional properties to food that might contribute to human health (94). Through a rational choice of raw material and starter culture, novel FF with improved functional properties that positively impact health can be generated (12, 67, 95). These novel FF can be produced using either a new species as a starter culture, a new raw material, or a combination of both. The European Commission defines foods as novel if they “had not been consumed to a significant degree by humans in the EU before 15 May 1997.” Interestingly, the current list of novel foods includes one fermented product, namely fermented wheat germ extract (96).

This systematic narrative review aims to evaluate the scientific evidence on whether novel FF confer health benefits to consumers. The research question addressed by this review will thus be: *“What are the health effects of novel fermented foods?”* The list of FF developed in the literature search string PIMENTO-LS will be combined with keywords relating to “novel food” and searched in combination with human studies. Studies related to “fermented wheat germ extract” will also be included. Given that this review leaves the investigated health conditions open, all primary and secondary endpoints reported by the publications will be extracted and evaluated. The evidence for potential health benefits of specific novel FF will be discussed in light of the significance and relevance of the reported endpoints. The review will identify research gaps that should foster future studies to promote innovation on novel FF and, eventually, their consumption (12).

OSF link to study protocol: PIMENTO-SP-S8.

### Food by-products

The increasing pressure on society to produce sustainable food systems has highlighted the importance of minimizing waste and making better nutritional use of by-products (97, 98). Whey, released in large amounts in the dairy processing industry, is an important milk by-product rich in proteins, lactose, minerals and vitamins (99). The nutrient composition of whey, particularly lactose and proteins, makes it a suitable source for fermentation by microorganisms. When fermented by *Lactobacillaceae* and *Bifidobacterium*, whey can be turned into a product with appealing texture and sensory properties (100). One of the most important beneficial health effects of fermented why is it enrichment in bioactive compounds compared to non-fermented whey. Indeed, *in vitro* and animal studies indicate that whey fermentation might increase its antioxidative properties (101). Several human studies reported using whey as a probiotic carrier to improve amino acid absorption in physically active males (102) or reduce food allergies (103, 104). Currently, no clear overview is available on the potential health-promoting effects of whey fermented by traditional methods, e.g., spontaneous fermentation or the addition of microorganisms as starters. This systematic narrative review will thus address the following question: *“What are the health effects of fermented whey?”*

Thus, this review aims to provide an overview on existing literature on the production, identification, and functional properties of fermented whey products. All human studies with fermented whey as part of their nutritional intake assessment will be included. Irrespective of the targeted health conditions, the primary and secondary endpoints will be extracted and analyzed for their significance and clinical relevance. Special attention will be given to characterization of the nutritional properties of fermented whey and differentiating it from unfermented whey to which probiotics were added. The review will identify research gaps that should foster future studies and promote innovation on fermented whey and, eventually, the consumption of fermented whey products beneficial for health.

OSF link to study protocol: PIMENTO-SP-S9.

## Discussion

### Towards a strategic research roadmap on the functionality of FF

A food company that aims at marketing a food product by claiming a specific health property, needs to document, and eventually grade, the strength of the available scientific evidence for the intended claim based on guidelines established by regulatory authorities such as the EFSA (13, 14). The body of evidence that should be provided for a successful claim encompasses the systematic reviewing of well designed, conducted, analyzed, and reported human studies (105). In particular, the PICO criteria (Population, Intervention, Control, Outcome) should be met, meaning that the characteristics of the enrolled participants, the investigated dietary components, the control, and the measured clinical endpoints should be in line with the research question underlying the claim. Furthermore, qualitatively good human studies should avoid the risk of bias arising from methodological flaws along the study workflow, including randomization bias, missing data, or selective reporting. In addition, the dietary components investigated should be well characterized with respect to their composition, stability, and safety. Finally, human studies reporting associations between dietary intake and clinical endpoints should be complemented with data (*in vitro*, animal, human) on the involved mechanism(s) of action in order to move the scientific evidence from associative to causative.

Whereas these regulatory guidelines primarily aim at protecting consumers, their role in promoting innovation for the industry is also recognized, although their efficacy has been debated (106–109). The food and nutrition research community could make use of regulatory guidelines (13, 14) and guidance for systematic reviewing of human studies (105) to improve the effectiveness of its pre-competitive research. Independently from their impact on innovation, these guidelines provide valuable information to researchers on design of studies that, when conducted and analyzed properly, will significantly contribute to the future evaluation of the impact of dietary components on health.

The U.S. Food and Drug Administration (FDA) recently announced a qualified health claim on yoghurt and diabetes, which states “Eating yogurt regularly may reduce the risk of type 2 diabetes according to limited scientific evidence” (110). This claim is interesting in the framework of this conceptual article for several reasons. Firstly, although submitted to the FDA by the private sector, the food product evaluated by the FDA is a generic yoghurt. In that regard, most of the data used to support the claim, in particular human studies, was produced by the academic research community, illustrating the role of public research in providing scientific evidence for the impact of food on health, in this particular case on the contribution of yoghurt to the reduction of type 2 diabetes risk. On the other hand, the researchers having conducted these studies did certainly not specifically design them to be part of the FDA health claim! Indeed, among the 66 human studies published on yoghurt and diabetes (20 intervention studies, 46 observation studies) only 28 observation studies were retained by the FDA. In particular, all of the intervention studies were excluded as they were not sufficiently controlled to provide useful information for assessment of this particular health claim. Also, risk of bias, e.g., due to improper baseline distribution of the participants or uncharacterized confounding factors, were put forwards to exclude 18 observation studies. These limitations led the FDA to downgrade their statement by qualifying the health claim with the words “may” before “reduce the risk of evidence” and “limited” before “scientific evidence.” We therefore argue here that guidance for health claims and the systematic reviewing of human studies could not only be used by researches to review and assess the strength of the available scientific evidence but also to draw lessons from the identified methodological weaknesses to propose scientifically more convincing studies in the future.

**Figure 1:**
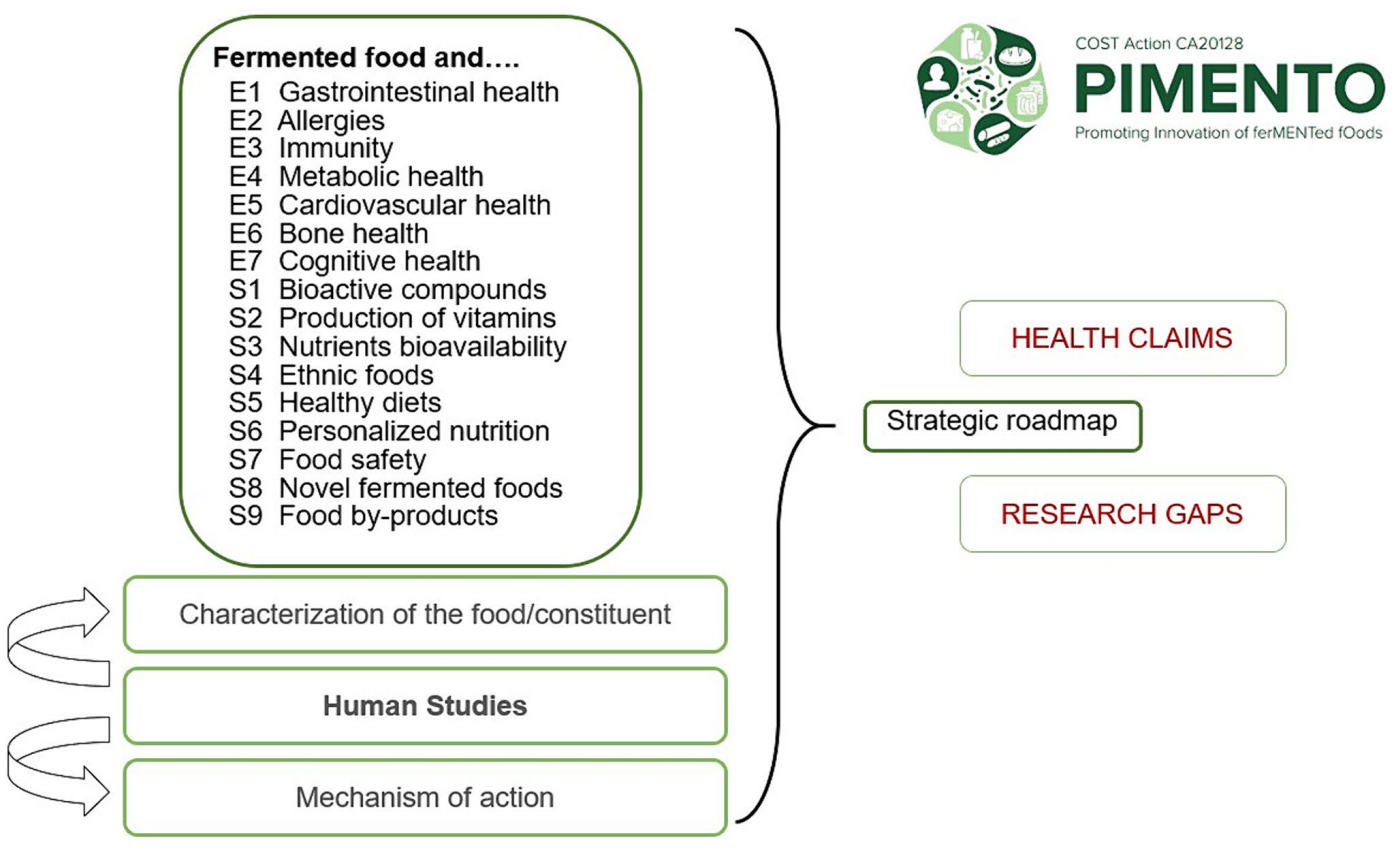
Overview of the reviewing strategy of PIMENTO WG3 to evaluate the health benefits and risks of fermented foods. E1–E7 refer to each of the seven “EFSA projects.” S1–S9 refer to each of the nine “Satellite projects.”

In conclusion, the systematic approach taken by PIMENTO WG3 to examine the 16 research questions will provide a novel comprehensive and harmonized array of findings that relate to the scientific evidence and the gaps in the research landscape on FF. This information will be used to define and publish a strategic roadmap for future research on the health benefits and risks of FF (Figure 1). Beyond the specific research questions addressed in each of the 16 reviews, the roadmap will define further research in the thematic areas identified at the Belgrade workshop. On a broader scale, the EFSA methodology used by PIMENTO WG3 to address the health benefits and risks of FF, which includes an evaluation of human studies, characterization of food components, and mechanistic data relevant to human health should be applied in the nutrition community beyond this food category.

## Supplementary material

Supplementary File 1 PIMENTO Study Protocol Template, PIMENTO-SP, used by each of the projects to fill and submit to OSF. The Study Protocols of each project have been deposited in OSF (links in text above) and the member area of the PIMENTO Website (https://fermentedfoods.eu/).

Supplementary File 2 PIMENTO Literature Search String, PIMENTO-LS, includes a search strategy of the literature in PubMed, Scopus, and the Cochrane Library Central.

## Glossary

BMI: Body mass index
BV: Bacterial vaginosis
CLA: Conjugated linoleic acid
CVD: Cardiovascular disease
EFSA: European Food Safety Authority
FA: Food allergy
FDA: U.S. Food and Drug Administration
FF: Fermented foods
GABA: Gamma-aminobutyric acid
GI: Gastrointestinal
HDL: High-density lipoprotein
HPA: Hypothalamic–pituitary–adrenal
LAB: Lactic acid bacteria
OSF: Open Science Framework
PIMENTO: Promoting innovation of fermented foods
RCT: Randomized clinical trial
SCFA: Short-chain fatty acid
T2D: Type 2 diabetes
VVC: Vulvovaginal candidiasis
WHO: World Health Organization
