# Correction: Health benefits and risks of fermented foods—the PIMENTO initiative

**DOI:** 10.3389/fnut.2025.1717279

**Published:** 2025-10-16

**Authors:** Frontiers Production Office

**Affiliations:** Frontiers Media SA, Lausanne, Switzerland

**Keywords:** fermented foods, food characterization, food safety, functional claims, health benefits, health claims, mechanism of action, systematic review

The conflict of interest statement was erroneously given as “The authors declare that the research was conducted in the absence of any commercial or financial relationships that could be construed as a potential conflict of interest. The author(s) declared that they were an editorial board member of Frontiers, at the time of submission. This had no impact on the peer review process and the final decision.”

The correct conflict of interest statement is “The authors declare that the research was conducted in the absence of any commercial or financial relationships that could be construed as a potential conflict of interest. The author(s) declared that they were an editorial board member of Frontiers, at the time of submission. This had no impact on the peer review process and the final decision. The handling editor MF is currently organizing a Research Topic with the author(s) BY, JD-S, AM.”

The original version of this article has been updated.

