# Supplementary material for: Health benefits and risks of fermented foods—the PIMENTO initiative
Source: Front Nutr. 2024 Sep 3;11:1458536. doi: 10.3389/fnut.2024.1458536 (PMC11414650; doi:10.3389/fnut.2024.1458536)
Supplement: Supplementary File 1 — PIMENTO Study Protocol Template, PIMENTO-SP, used by each of the projects to fill and submit to OSF. The Study Protocols of each project have been deposited in OSF (links in text above) and the member area of the PIMENTO Website (https://fermentedfoods.eu/). [file Presentation_1.pdf]

PIMENTO STUDY PROTOCOL-XX (version 1.7.2024)

Preamble – This study protocol is one among the 16 study protocols of reviews conducted by the working group 3 (WG3) of the COST Action CA20128 - Promoting Innovation of Fermented Foods (PIMENTO). These reviews summarize the evidence for a set of 16 functional properties of fermented foods. WG3 reviews follow the guidance of the European Food Safety Authority (EFSA) “Scientific and technical guidance for the preparation and presentation of a health claim application” (EFSA Panel on Dietetic Products, 2021b). This guidance is composed of three major parts: (i) a systematic review of human studies; (ii) a non-systematic reviewing of the characteristics of the investigated fermented foods; (iii) a non-systematic reviewing of evidence supporting the functional properties of the investigated fermented foods, in particular the mechanism of action and the bioaccessibility and bioavailability of the active compounds. In addition, safety aspects will also be considered in the reviews.
The systematic reviewing of the human studies is conducted based on the guidance by Muka *et al.* (Muka et al., 2020). The PROSPERO study protocol is used as basis to structure the reviewing process of the human studies. It is also adapted to the needs of the WG3 reviews to include the non-systematic parts of the reviews described above to create this generic PIMENTO Study Protocol (PIMENTO-SP). Each of the sections below contains a generic methodology valid for all reviews. The PIMENTO-SP, however, allows for the introduction of review-specific information and methodology. The study protocols of each of the 16 WG3 reviews are deposited in Open Science Framework Registries (OSF) (https://osf.io/registries) using the Open-Ended-Registration modus (Bakker et al., 2020). The narrative summary of the review also deposited in OSF is a copy-paste of the paragraph describing the review in the PIMENTO position paper by *Todorovic et al*. to which this study protocol is linked.
This study protocol (PIMENTO-SP-XX) describes the workflow aimed at reviewing text. The review based on this study protocol uses the EFSA guidance [title and reference].

1. Review title

Ideally the title should state succinctly the interventions or exposures being reviewed and the associated health or social problems. Where appropriate, the title should use the PI(E)COS structure to contain information on the Participants, Intervention (or Exposure) and Comparison groups, the Outcomes to be measured and Study designs to be included.

Provide title.

2. Original language

English

3. Anticipated or actual start date

The date of commencement for the systematic review can be defined as any point after completion of a protocol but before formal screening of the identified studies against the eligibility criteria begins.

1.10.2023

4. Anticipated completion date

31.10.2025

5. Stage of review at time of this submission

Indicate the stage of progress of the review by ticking the relevant Started and Completed boxes. Please note: Reviews that have progressed beyond the point of completing data extraction at the time of initial registration are not eligible for inclusion in the study protocol. Should evidence of incorrect status and/or completion date being provided at the time of submission come to light, the content of the study protocol will be removed leaving only the title and named contact details and a statement that inaccuracies in the

stage of the review date had been identified. This field should be updated when any amendments are made to a published record and on completion and publication of the review.

Review stage	Started	Completed
Preliminary searches	Yes	No
Piloting of the study selection process	Yes	No
Formal screening of search results against eligibility criteria	No	No
Data extraction	No	No
Risk of bias (quality) assessment	No	No
Data analysis	No	No

See sections 23-25 for reviews-specific adaptation of the review process.

6. Contact persons

Name, mail, address, phone number, and affiliation of project leaders:
Text.

7. Review team members and their organizational affiliations

The review team members listed below *have already* contributed and plan to further contribute to the review. Those persons will be co-authors of the manuscript, based on their current and future contribution to each of the following three tasks. The table will be adapted as the work progress.

1. Planning and contribution to one or several components (conception, design, conduct, analysis, or interpretation) of the work which led to the paper or interpreting at least a portion of the results;
2. Writing a draft of the article or revising it for intellectual content; and
3. Final approval of the version to be published.

First name	Last name	Affiliation
…	…	…
…	…	…
…	…	…

8. Funding sources/sponsors

Details of the individuals, organizations, groups or other legal entities who take responsibility for initiating, managing, sponsoring and/or financing the review.

This review is part of the COST Action CA20128 - Promoting Innovation of Fermented Foods (PIMENTO).

9. Conflicts of interest

The conflicts of interest listed cover the review team as a whole, as well as individuals in the team:
Write either “The authors declare no conflict of interest” or describe the conflict(s) of interest.

10. Collaborators

All contributors to the review are listed under point 7.

11. Review question

Review-specific text:
Text.

Systematic review of human studies (sections 12-26)

12. Searches

Databases: PubMed, Scopus, Cochrane Library
Dates: 1.1.1970 – 31.8.2023.
Language: English

13. Search strategy

The generic search strings developed by the Library of the University of Zurich (Alisa Berger) for PIMENTO will be used by each of the 16 reviews (PIMENTO search strings). These strings encompass terms for the search of a broad scope of fermented foods across all food groups, of all types of human studies, as well as of dietary intake. The generic search string will be published as Supplementary file in the position paper by Todorovic *et al*. to which is study protocol is linked.

The complete search (strings) for each Project will be published as Supplementary file of the review.

In addition to the PIMENTO search strings, review-specific strings covering the search for the functional part of the review (e.g., clinical indication, biological activity) will be defined as specified below. Review-specific changes from the PIMENTO search strings will be presented and justified in the manuscript.

Specify the functional part of the review (e.g., clinical indication, biological activity):
Text.

Present and justify review-specific changes from the PIMENTO search strings:
Provide information or write "No review-specific changes".

14. Condition or domain being studied

Give a short description of the disease, condition, or healthcare domain being studied:
Text.

Of note the manuscript of the reviews will contain a description of the condition being studied based on Section 3 "Characterization of the claimed effect" of the EFSA guidance (EFSA Panel on Dietetic Products, 2021b).

15. Participants/population

Summary criteria for the participants or populations being studied by the review. The preferred format includes details of both inclusion and exclusion criteria. Example: Inclusion: Adults with schizophrenia (as diagnosed using any recognised diagnostic criteria). Exclusion: Adolescents (under 18 years of age) and elderly people (over 70).

Adults are considered from the age of 18 and above.

Inclusion: Text.
Exclusion: Text.

16. Intervention(s), exposure(s)

Full and clear descriptions or definitions of the nature of the interventions or the exposures to be reviewed. Report the intervention/exposure in enough detail that others could reproduce it or assess its applicability to their own setting. The preferred format includes details of both inclusion and exclusion criteria. For reviews of qualitative studies give details of the focus of the review.

The intervention/exposure consists of the ingestion of any of the fermented foods contained in the PIMENTO search string for fermented foods across the following food groups: dairy, meat and fish, fruits

and vegetables, beverages, legumes, cereals and grains. Alcoholic beverages with an alcohol content of more than 1.25% will be excluded. Unless specified otherwise, no limits are set for duration or dosage of the ingested fermented food(s). Unless specified otherwise, studies investigating application of fermented foods other than for nutritional purpose (e.g., nasal or topical) will be excluded. In addition, studied investigating probiotics will be excluded unless the probiotics is/are added at the beginning of the fermentation process and that there are indications from the literature that the probiotic strain(s) contribute(s) to the fermentation of the food matrix. Interventions including any possible confounders such as prebiotic fibres or added bioactive compounds will not be included. Intervention could be designed as a stand-alone intervention or as a combined intervention if the comparator conditions are adequately controlled for non-fermented interventions.

Present and justify review-specific changes from the above guidance. In particular specify the food groups or foods from the PIMENTO search string if the review restricts the search to a subset of fermented foods or food groups. Provide also supplementary information as needed, in particular to define additional inclusion or exclusion criteria:
Provide information or write “No review-specific changes”.

17. Comparator(s)/control

The control could be the absence of consumption, or consumption of a lower amount or lower frequency of the fermented food/diet of interest or the consumption of a corresponding non-fermented food/diet. Any adequate non-fermented placebo or control (such as another medication or treatment) will also be accepted as a valid comparator.

The definition of appropriate controls is an important problem in nutritional science. In order to highlight research gaps on this issue as well as to collect data from all human studies as described in the EFSA guidance (EFSA Panel on Dietetic Products, 2021a, 2021b) human studies will first be selected for evaluation irrespective of the quality of the control. The quality of the control will be evaluated subsequently for all studies meeting the PI(E)O criteria (Population, Intervention/Exposure, Outcome).
Provide information or write “No review-specific supplementary information”.

18. Types of study to be included

All human studies will be searched systematically and included according to article 4.2.1. of the EFSA guidance (EFSA Panel on Dietetic Products, 2021b):

- Publications reporting on human intervention (efficacy) studies (e.g., randomised controlled studies, randomised uncontrolled studies, non-randomised controlled studies, other intervention studies)
- Publications reporting on human observational studies (e.g., cohort studies, case-control studies, cross-sectional studies, other observational studies)
- Available systematic reviews with or without meta-analysis will be used to check for potentially missing studies.

Animal and *in vitro* studies will be excluded from the systematic reviewing process.

Present and justify review-specific changes from the above guidance. Also, provide supplementary information, as needed, in particular to define additional inclusion or exclusion criteria:
Provide information or write “No review-specific changes”.

19. Context

Present, as needed, inclusion/exclusion criteria not described elsewhere:
Provide information or write “No additional criteria”.

20. Main outcome(s)

Give the pre-specified primary (most important) outcomes of the review, including details of how the outcome is defined and measured and when these measurements are made, if these are part of the review inclusion criteria. For systematic reviews of qualitative studies give details of what the review aims to achieve:
Text.

21. Additional outcome(s)

List the pre-specified secondary (additional) outcomes of the review, with a similar level of detail to that required for primary outcomes:
Provide information or write “No additional outcome”.

If available, an evaluation of the contribution of the gut microbiota to the main or additional outcomes will be presented.

In addition, outcomes related to safety (e.g., adverse effects or risk markers) will be recorded as indicated in Appendix B of the EFSA guidance (EFSA Panel on Dietetic Products, 2021b).

22. Data extraction (selection and coding)

Study selection and data extraction will be conducted based on the following steps of the guidance of Muka *et al.*(2020): steps 4 (Define selection criteria), 5 (Design data collection form), 8 (Collection of references and abstracts in a single file), 9 (Elimination of duplicates), 10 (Screening of the titles and abstracts by at least two reviewers), 11 (Collection, comparison, and selection of references for retrieval), 12 (Retrieval of full text and application of selection criteria), 13, if needed (Contact experts), 14 (Search for additional references), 16 (Application of the data collection form), and 18 (Preparation of the database for analysis).

The data extraction form(s) of the interventional and observational studies will be based on combined information provided in the handbook of the Cochrane interactive learning course “Conducting an Intervention Review” (Higgins et al., 2022), Appendix B “Information to be presented in a full study report for human efficacy studies” of the EFSA guidance (EFSA Panel on Dietetic Products, 2021b) and the STROBE guidelines for reporting observational studies (von Elm et al., 2007), respectively. CADIMA software (Kohl et al., 2018) will be used to select the references. A consistency test will be performed in CADIMA using a subset of the literature dataset and, if needed, the study protocol and/or the literature selection strategy will be adapted to improve the efficacy and systematicity of the reviewing process. Overview of the selection process to identify the relevant studies will be documented with a flow diagram. The references will be screened based on titles, abstracts, and texts.

23. Risk of bias (quality) assessment

The risk of bias will be conducted based on steps 17 (Evaluate study quality and risk of bias), 21 (Exploration of heterogeneity), and 22 (Check reporting bias) of Muka *et al.* (2020) using tools, such as ROBINS.

Provide supplementary information, as needed
Write either “The review will be narrative. Accordingly, the data synthesis will focus on a description of the clinical indications as well as primary and secondary endpoints and bias will not be evaluated” or “No review-specific supplementary information” or provide review-specific information as needed.

24. Strategy for data synthesis

Data synthesis will be based on steps 19 (Conduct descriptive synthesis) and 23 (Check the quality of the evidence: the confidence in the results presented) of Muka *et al.* (2020).

An evaluation of the quality of the evidence derived from the human studies will be conducted according to the Grading of Recommendations Assessment (GRADE) according to Module 7 (Interpreting the

findings) of the handbook of the Cochrane interactive learning course “Conducting an Intervention Review” (Higgins et al., 2022).

In case a sufficient number of relevant human studies is available, a quantitative analysis (meta-analysis) of the data will be conducted using appropriate statistical approaches according to Module 6 (Analysing the data) of the handbook of the Cochrane interactive learning course “Conducting an Intervention Review” (Higgins et al., 2022).

Present and justify review-specific changes from the above guidance. Also, provide supplementary information, as needed:
Write either “The review will be narrative. Accordingly, the data synthesis will focus on a description of the clinical indications as well as primary and secondary endpoints.” or “No review-specific changes” or provide review-specific information as needed.

25. Analysis of subgroups or subsets

Analysis of heterogeneity will be based on step 21 (Exploration of heterogeneity) of Muka *et al.* (2020). If appropriate subgroup analyses will be conducted according to Module 6 (analysing the data) of the handbook of the Cochrane interactive learning course “Conducting an Intervention Review” (Higgins et al., 2022).

Present and justify review-specific changes from the above guidance. Also, provide supplementary information, as needed:
Write either “The review will be narrative. Accordingly, the data synthesis will focus on a description of the clinical indications as well as primary and secondary endpoints and a quantitative analysis of subgroups will not be conducted” or “No review-specific changes” or provide review-specific information as needed.

26. Type and method of review

Mention below the type of review of the human studies:
Write “Systematic review” OR “Systematic narrative review” OR “Systematic scoping review” OR “Scoping review”.

Health area of the review (for example based on the health area proposed by the PROSPERO study protocol):
Text.

Non-systematic part of the review (sections 27-29)

As already presented in the preamble, the systematic review of the available human data described in the above sections 12-26 will be complemented with a non-systematic reviewing of the literature that includes the characteristics of the investigated fermented food(s) as well evidence supporting the functional properties of the investigated fermented food(s) according to the EFSA guidance (EFSA Panel on Dietetic Products, 2021b) and safety issues.

27. Characteristics of the investigated fermented foods

Description of the characterization of the food constituent (nutrient, microorganism, food, diet) according to Section 2 of the EFSA guidance (Characterization of the food/constituent). The literature search for this part will depend on the fermented foods used for the relevant human studies identified.

Present and justify review-specific changes from the above guidance. Also, provide supplementary information, as needed:
Write either “No review-specific changes” or provide review-specific information as needed.

28. Evidence supporting the functional properties of the investigated fermented foods

Descriptive analysis of the supportive evidence for the research question based on human, animal and *in vitro* studies according to Section 5.2.3 of the EFSA guidance (Supportive evidence), in particular mechanism of action and bioavailability/bioaccessibility. If available an evaluation of the contribution of the gut microbiota (animal model; human studie) to the main or additional outcomes will be presented.

Present and justify review-specific changes from the above guidance. Also, provide supplementary information, as needed:
Write either "No review-specific changes" or provide review-specific information as needed.

29. Safety of fermented foods

Safety of the fermented foods analysed and discussed critically by considering data from the human studies selected for the systematic reviewing, complemented with additional relevant human, animal, and *in vitro* studies.

Present and justify review-specific changes from the above guidance. Also, provide supplementary information, as needed:
Write either "No review-specific changes" or provide review-specific information as needed.

Summary of the systematic and non-systematic parts of the review (section 30)

If appropriate, the functional effects of the fermented food based on the totality of the evidence will be evaluated according to Section 1 of the EFSA guidance (Technical dossier).

30. Summary of the evidence

Analysis of the clinical data according to guidance provided in Section 5 of the EFSA guidance (Overall summary of pertinent scientific data) in particular according to Sections 5.2.1 (Substantiation of a causal relationship between the consumption of the food/constituent and the claimed effect) and 5.2.2. (Characterisation of the relationship between the consumption of the food/constituent and the claimed effect).

The evidence for a functional property of the fermented food(s) of interest will be evaluated according to the Summary section of the EFSA guidance for health claims (EFSA Panel on Dietetic Products, 2021b) that takes into account the totality of the available scientific data subject to the specific conditions of use. The totality of the evidence includes in particular the quality of the characterization of the food/constituent, the evidence for the biological effect of the reviewed fermented food(s), whether a cause and effect relationship has been established between the consumption of the food/constituent and the biological effect in humans, as wells as whether the quantity of the food/constituent and pattern of consumption required to obtain the biological effect could reasonably be achieved as part of a balanced diet.

The outcome of the scientific assessment of the evidence will be worded according to the EFSA guidance "General scientific guidance for stakeholders on health claim Applications" (EFSA Panel on Dietetic Products, 2021a) with one of the following three formulations:

i. A cause and effect relationship has been established between the consumption of the food/constituent and the claimed effect.
ii. The evidence provided is insufficient to establish a cause-and-effect relationship between the consumption of the food/constituent and the claimed effect.
iii. A cause-and-effect relationship has not been established between the consumption of the food/constituent and the claimed effect.

Present and justify review-specific changes from the above guidance. Also, provide supplementary information, as needed:
Write either "No review-specific changes" or provide review-specific information to as needed.

31. Language

English

32. Country

List the countries of all co-authors:
Text.

The list will be adapted as the work progresses.

33. Other registration details

The protocol will be deposited in Open Science Framework (OSF) and published on the webpage of PIMENTO.

34. Reference and/or URL for published protocol

Open Science Framework reference:
Provide OSF reference.

The project co-leaders give permission for this file to be made publicly available before the review is completed: YES

35. Dissemination plans

The manuscript will be submitted for publication, as one of a series of sixteen PIMENTO review articles. These articles will be preceded by a position paper published in the same journal as the one to which this study protocol is linked (Todorovic et al.). Publication of the sixteen review articles will be followed by the publication, in the same journal to which this study protocol is linked, of a strategic roadmap for future research on the health benefits and risks of fermented foods. The co-authors of the 16 EFSA and Satellite reviews will also be proposed to contribute as co-authors of the strategic roadmap.

Dissemination of information derived from this review will be conducted according to PIMENTO policy.

36. Keywords

Give words or phrases that best describe the review:
Fermented foods; Text for additional keywords.

37. Details of any existing review of the same topic by the same authors

Give details of earlier versions of *this* systematic review if an update of an existing review is being registered, including full bibliographic reference if possible:
Provide reference(s) or write “None”.

38. Current review status

Status:
Write “Ongoing” or “Completed”.

39. Details of final report/publication(s)

Provide information once the review is published:
Text.

40. References

Bakker, M., Veldkamp, C.L.S., van Assen, M., Crompvoets, E.A.V., Ong, H.H., Nosek, B.A., Soderberg, C.K., Mellor, D., Wicherts, J.M., 2020. Ensuring the quality and specificity of preregistrations. PLoS Biol 18(12), e3000937.

EFSA Panel on Dietetic Products, N.a.A., 2021a. General scientific guidance for stakeholders on health claim applications (Revision 1). Efsa j 19(3), e06553.

EFSA Panel on Dietetic Products, N.a.A., 2021b. Scientific and technical guidance for the preparation and presentation of a health claim application (Revision 3). Efsa j 19(3), e06554.

Higgins, J., Thomas, J., Chandler, J., Cumpston, M., Li, T., Page, M., Welch, V., 2022. Cochrane Handbook for Systematic Reviews of Interventions version 6.3 (updated February 2022)Available from www.training.cochrane.org/handbook. www.training.cochrane.org/handbook.

Kohl, C., McIntosh, E.J., Unger, S., Haddaway, N.R., Kecke, S., Schiemann, J., Wilhelm, R., 2018. Online tools supporting the conduct and reporting of systematic reviews and systematic maps: a case study on CADIMA and review of existing tools. Environmental Evidence 7(1), 8.

Muka, T., Glisic, M., Milic, J., Verhoog, S., Bohlius, J., Bramer, W., Chowdhury, R., Franco, O.H., 2020. A 24-step guide on how to design, conduct, and successfully publish a systematic review and meta-analysis in medical research. Eur J Epidemiol 35(1), 49-60.

von Elm, E., Altman, D.G., Egger, M., Pocock, S.J., Gøtzsche, P.C., Vandenbroucke, J.P., 2007. Strengthening the Reporting of Observational Studies in Epidemiology (STROBE) statement: guidelines for reporting observational studies. Bmj 335(7624), 806-808.

Provide additional references, as needed:
Text.

41. Revisions of previous versions of this document

Section 17 “Comparator(s)/control” has been adapted to take into account the fact that the selection of the studies according to the PICO criteria, was a two-step procedure in which the studies were first screened according to PIO criteria. The retained studies were then evaluated for the C criteria, allowing the review to report on the quality of the controls in the study selection.

Additional changes:
Write ‘None’ or present the changes made, the rationale for the changes, and the date of the previous version.
