# Supplementary material for: Health benefits and risks of fermented foods—the PIMENTO initiative
Source: Front Nutr. 2024 Sep 3;11:1458536. doi: 10.3389/fnut.2024.1458536 (PMC11414650; doi:10.3389/fnut.2024.1458536)
Supplement: Supplementary File 2 — PIMENTO Literature Search String, PIMENTO-LS, includes a search strategy of the literature in PubMed, Scopus, and the Cochrane Library Central. [file Data_Sheet_1.pdf]

Supplementary File 2. Search stings for the search of published human studies related to the health benefits and risks of fermented foods (PIMENTO-LS). The words “OR” in larger font and bold separate different food groups

<u>Medline search (PubMed)</u>

#1 "Fermented Foods"[Mesh] OR "Fermentation"[Mesh] OR ("Food"[Mesh] AND ferment*[tiab]) OR ((ferment*[tiab] OR cultur*[tiab] OR leaven*[tiab]) AND (food*[tiab] OR drink*[tiab] OR beverage*[tiab])) OR "Fermented product"[tiab:~6] OR "Fermented products"[tiab:~6] OR "cultured product"[tiab:~6] OR "cultured products"[tiab:~6] OR "product fermentation"[tiab:~6] OR "products fermentation"[tiab:~6] OR "starter culture*"[tiab] **OR** ((ferment*[tiab] OR culture*[tiab] OR sour*[tiab]) AND (milk[tiab] OR dairy[tiab])) OR buttermilk[tiab] OR sour cream*[tiab] OR cheese*[tiab] OR yoghurt[tiab] OR yogurt[tiab] OR "yoghourt"[tiab] OR "yakult"[tiab] OR "quark"[tiab] OR "kefir"[tiab] OR "lassi"[tiab] OR "kumis"[tiab] OR "koumiss"[tiab] OR "kajmak"[tiab] OR "airag"[tiab] OR "ayran"[tiab] OR "calpis"[tiab] OR "borhani"[tiab] OR "chal"[tiab] OR "doogh"[tiab] OR kvass[tiab] OR skyr[tiab] OR amasi[tiab] OR bouza[tiab] OR butter*[tiab] OR chal[tiab] OR filmjolk[tiab] OR kishk[tiab] OR labne*[tiab] **OR** ((Ferment*[tiab] OR cultur*[tiab] OR cured[tiab]) AND (meat*[tiab] OR fish*[tiab] OR seafood*[tiab] OR shellfish[tiab] OR sausage*[tiab])) OR "salami"[tiab] OR "pepperoni"[tiab] OR peperoni[tiab] OR "chorizo"[tiab] OR "cervelat"[tiab] OR "mettwurst"[tiab] OR "summer sausage"[tiab] OR "sucuk"[tiab] OR "dried meat"[tiab] OR "dried sausage"[tiab] OR "dry sausage"[tiab] OR "fish sauce"[tiab] OR "shrimp paste"[tiab] OR "shrimp sauce"[tiab] OR "oyster sauce"[tiab] OR "prosciutto"[tiab] OR "pancetta"[tiab] OR "saucisson"[tiab] OR sucuk[tiab] **OR** ((Ferment*[tiab] OR cultur*[tiab]) AND (fruit*[tiab] OR vegetable*[tiab] OR coconut*[tiab] OR almond*[tiab] OR hazelnut*[tiab] OR nut[tiab] OR cucumber*[tiab] OR lemon*[tiab] OR citrus[tiab] OR cabbage*[tiab] OR cauliflower*[tiab] OR pepper*[tiab] OR carrot*[tiab] OR olive*[tiab] OR onion*[tiab] OR sago[tiab])) OR "sauerkraut"[tiab] OR "table olive"[tiab] OR pickle*[tiab] OR "kimchi"[tiab] OR "paocai"[tiab] OR torshi[tiab] **OR** ((Ferment*[tiab] OR cultur*[tiab]) AND (tea[tiab] OR teas[tiab] OR juice*[tiab])) OR "beer"[tiab] OR "wine"[tiab] OR "cider*"[tiab] OR shochu[tiab] OR "kombucha*"[tiab] OR "pulque"[tiab] OR puer[tiab] OR pu’er[tiab] OR pu-er[tiab] OR pu-erh[tiab] OR "pu erh"[tiab] OR "fuzhuan"[tiab] OR "dark tea*"[tiab] OR "yellow tea*"[tiab] OR coffee[tiab] OR shalgam[tiab] OR hardaliye[tiab] **OR** ((Ferment*[tiab] OR cultur*[tiab]) AND (soy[tiab] OR soya[tiab] OR bean*[tiab] OR pea[tiab] OR peas[tiab] OR lentil*[tiab] OR chickpea*[tiab] OR legume*[tiab] OR pulse*[tiab] OR (poi[tiab])) OR "soy sauce"[tiab] OR "soya sauce"[tiab] OR "soybean paste"[tiab] OR "miso"[tiab] OR "tempeh"[tiab] OR tempe[tiab] OR "natto"[tiab] OR "doenjang"[tiab] OR "doubanjiang"[tiab] OR douchi[tiab] OR "gochujang"[tiab] OR cheonggukjang[tiab] OR tsukemono[tiab] OR garri[tiab] **OR** ((Ferment*[tiab] OR cultur*[tiab] OR leaven*[tiab]) AND (cereal*[tiab] OR grain*[tiab] OR wheat*[tiab] OR oat[tiab] OR oats[tiab] OR rice*[tiab] OR millet*[tiab] OR sorghum*[tiab] OR maize*[tiab] OR rye[tiab] OR barley*[tiab] OR chia[tiab] OR oilseed*[tiab] OR teff[tiab])) OR "bread"[tiab] OR "sourdough"[tiab] OR "crispbread"[tiab] OR "boza"[tiab] OR "ogi"[tiab] OR dosa[tiab] OR "tarhana"[tiab] OR "buckwheat"[tiab] OR "spelt"[tiab] OR "einkorn"[tiab] OR "quinoa"[tiab] OR "amaranth"[tiab] OR "tef"[tiab] OR "bushera"[tiab] OR chica[tiab] OR chicha[tiab] OR choujiu[tiab] OR injera[tiab] OR mahewu[tiab] OR ogiri[tiab] OR pozol[tiab] OR ugba[tiab]) **OR** ((Ferment*[tiab] OR cultur*[tiab]) AND ("condiment*"[tiab] OR relish*[tiab] OR horseradish[tiab] OR "dressing*"[tiab] OR "seasoning*"[tiab] OR "sauce*"[tiab] OR cocoa*[tiab] OR tuber[tiab] OR "acetic acid"[tiab])) OR "chocolate*"[tiab] OR "vinegar*"[tiab] OR "tabasco"[tiab] OR "sriracha"[tiab] OR "Worcestershire"[tiab] OR "Worcester"[tiab]

#2 "Diet"[Mesh] OR "Life Style"[Mesh] OR "Eating"[Mesh] OR "Feeding Behavior"[Mesh] OR ((food[tiab] OR macronutrient*[tiab] OR eating[tiab]) AND (intake*[tiab] OR habit*[tiab] OR behavior*[tiab] OR pattern*[tiab])) OR diet*[tiab] OR intake[tiab] OR ingestion[tiab] OR suppl*[tiab] OR consumption[tiab] OR meal*[tiab] OR nutrient*[tiab] OR nutrit*[tiab]

#3 Functional strings specific to each PIMENTO WG3 review

#4 #1 AND #2 AND #3

#5 "Diet Surveys"[Mesh] OR "Cohort Studies"[Mesh] OR cohort*[Tiab] OR prospective[Tiab] OR longitudinal[Tiab]

#6 Randomized Controlled Trial[Publication Type] OR Controlled Clinical Trial[Publication Type] OR Pragmatic Clinical Trial[Publication Type] OR Clinical Study[Publication Type] OR Adaptive Clinical Trial[Publication Type] OR Equivalence Trial[Publication Type] OR Clinical Trial[Publication Type] OR Clinical Trial, Phase I[Publication Type] OR Clinical Trial, Phase II[Publication Type] OR Clinical Trial, Phase III[Publication Type] OR Clinical Trial, Phase IV[Publication Type] OR Clinical Trial Protocol[Publication Type] OR multicenter study[Publication Type] OR "Clinical Studies as Topic"[Mesh] OR "Clinical Trials as Topic"[Mesh] OR "Clinical Trial Protocols as Topic"[Mesh] OR "Multicenter Studies as Topic"[Mesh] OR "Random Allocation"[Mesh] OR "Double-Blind Method"[Mesh] OR "Single-Blind Method"[Mesh] OR "Placebos"[Mesh:NoExp] OR "Control Groups"[Mesh] OR "Cross-Over Studies"[Mesh] OR random*[Title/Abstract] OR sham[Title/Abstract] OR placebo*[Title/Abstract] OR ((singl*[Title/Abstract] OR doubl*[Title/Abstract]) AND (blind*[Title/Abstract] OR dumm*[Title/Abstract] OR mask*[Title/Abstract])) OR ((tripl*[Title/Abstract] OR trebl*[Title/Abstract]) AND (blind*[Title/Abstract] OR dumm*[Title/Abstract] OR mask*[Title/Abstract])) OR "control study"[tiab:~3] OR "control studies"[tiab:~3] OR "control group"[tiab:~3] OR "control groups"[tiab:~3] OR "healthy volunteers"[tiab:~3] OR "control trial"[tiab:~3] OR "control trials"[tiab:~3] OR "controlled study"[tiab:~3] OR "controlled trial"[tiab:~3] OR "controlled studies"[tiab:~3] OR "controlled trials"[tiab:~3] OR "clinical study"[tiab:~3] OR "clinical studies"[tiab:~3] OR "clinical trial"[tiab:~3] OR "clinical trials"[tiab:~3] OR Nonrandom*[Title/Abstract] OR non random*[Title/Abstract] OR non-random*[Title/Abstract] OR quasi-random*[Title/Abstract] OR quasirandom*[Title/Abstract] OR "phase study"[tiab:~3] OR "phase studies"[tiab:~3] OR "phase trial"[tiab:~3] OR "phase trials"[tiab:~3] OR "crossover study"[tiab:~3] OR "crossover studies"[tiab:~3] OR "crossover trial"[tiab:~3] OR "crossover trials"[tiab:~3] OR "cross-over study"[tiab:~3] OR "cross-over studies"[tiab:~3] OR "cross-over trial"[tiab:~3] OR "cross-over trials"[tiab:~3] OR ((multicent*[tiab] OR multi-cent*[tiab] OR open label[tiab] OR open-label[tiab] OR equivalence[tiab] OR superiority[tiab] OR non-inferiority[tiab] OR noninferiority[tiab] OR quasiexperimental[tiab] OR quasi-experimental[tiab]) AND (study[tiab] OR studies[tiab] OR trial*[tiab])) OR allocated[tiab] OR pragmatic study[tiab] OR pragmatic studies[tiab] OR pragmatic trial*[tiab] OR practical trial*[tiab].

#7 "Epidemiologic Methods"[Mesh:NoExp] OR "Epidemiologic Studies"[Mesh] OR "Observational Studies as Topic"[Mesh] OR "Clinical Studies as Topic"[Mesh] OR "Single-Case Studies as Topic"[Mesh] OR "Organizational Case Studies"[Mesh] OR observational study[Publication Type] OR validation study[Publication Type] OR clinical study[Publication Type] OR case reports[Publication Type] OR "observational study"[tiab:~3] OR "observational studies"[tiab:~3] OR "observational design"[tiab:~3] OR "observational analysis"[tiab:~3] OR "observational analyses"[tiab:~3] OR ((cohort*[tiab] OR prospective[tiab] OR follow-up[tiab] OR longitudinal[tiab] OR long-term[tiab] OR retrospective[tiab]) AND (study[tiab] OR studies[tiab] OR design[tiab] OR analysis[tiab] OR analyses[tiab] OR data[tiab] OR review[tiab])) OR case control*[tiab] OR case comparison*[tiab] OR case-referent[tiab] OR "population study"[tiab:~3] OR "population studies"[tiab:~3] OR "population analysis"[tiab:~3] OR "population analyses"[tiab:~3] OR "descriptive study"[tiab:~3] OR "descriptive studies"[tiab:~3] OR "descriptive design"[tiab:~3] OR "descriptive analysis"[tiab:~3] OR "descriptive analyses"[tiab:~3] OR "multidimensional study"[tiab:~3] OR "multidimensional studies"[tiab:~3] OR "multidimensional design"[tiab:~3] OR "multidimensional analysis"[tiab:~3] OR "multidimensional analyses"[tiab:~3] OR "cross-sectional study"[tiab:~3] OR "cross-sectional studies"[tiab:~3] OR "cross-sectional design"[tiab:~3] OR "cross-sectional analysis"[tiab:~3] OR "cross-sectional analyses"[tiab:~3] OR "cross-sectional research"[tiab:~3] OR "cross-sectional survey"[tiab:~3] OR "cross-sectional findings"[tiab:~3] OR natural experiment*[tiab] OR quasi experiment*[tiab] OR "nonexperimental study"[tiab:~3] OR "nonexperimental studies"[tiab:~3] OR "nonexperimental design"[tiab:~3] OR "nonexperimental analysis"[tiab:~3] OR "nonexperimental analyses"[tiab:~3] OR "prevalence study"[tiab:~3] OR "prevalence studies"[tiab:~3] OR "prevalence analysis"[tiab:~3] OR "prevalence analyses"[tiab:~3] OR case series[tiab] OR "case report"[tiab:~3] OR "case reports"[tiab:~3] OR "case study"[tiab:~3] OR "case studies"[tiab:~3] OR "case histories"[tiab:~3]

#8 "systematic review"

#9 #5 OR #6 OR #7 OR #8

#10 #4 AND #9

#11 #10 NOT (("Child"[Mesh] OR "Infant"[Mesh] OR "Adolescent"[Mesh]) NOT "Adult"[Mesh])

#12 #11 NOT (("Animals"[Mesh] OR "Animal Experimentation"[Mesh] OR "Models, Animal"[Mesh] OR "Vertebrates"[Mesh]) NOT ("Humans"[Mesh] OR "Human Experimentation"[Mesh]))

#13 #12 NOT ("Breast Feeding"[Majr] OR "Milk, Human"[Majr])

#14 #13 AND (English[Filter])

#15 #14 AND (("1970/01/01"[Date - Publication] : "2023/08/31"[Date - Publication]))

Scopus search

#1 TITLE-ABS-KEY ((ferment* OR cultur* OR leaven*) W/6 (food* OR drink* OR beverage*) OR "starter culture*") OR TITLE-ABS-KEY ((ferment* OR cultur* OR leaven*) W/2 product*) **OR** TITLE-ABS-KEY (((ferment* OR culture* OR sour*) W/6 (milk OR dairy OR cream* OR quark)) OR buttermilk OR cheese* OR yoghurt OR yogurt OR yoghourt OR yakult OR kefir OR lassi OR kumis OR koumiss OR kajmak OR airag OR ayran OR calpis OR borhani OR chal OR doogh OR kvass OR skyr OR amasi OR bouza OR butter* OR chal OR filmjolk OR kishk OR labne*) **OR** TITLE-ABS-KEY (((Ferment* OR cultur* OR cured) W/6 (meat* OR fish* OR seafood* OR shellfish OR sausage*)) OR "salami" OR "pepperoni" OR peperoni OR "chorizo" OR "cervelat" OR "mettwurst" OR "summer sausage" OR "sucuk" OR "dried meat*" OR "dried sausage*" OR "dry sausage*" OR "fish sauce*" OR "shrimp paste" OR "shrimp sauce" OR "oyster sauce" OR "prosciutto" OR "pancetta" OR "saucisson" OR sucuk) **OR** TITLE-ABS-KEY (((Ferment* OR cultur*) W/6 (fruit* OR vegetable* OR coconut* OR almond* OR hazelnut* OR nut OR cucumber* OR lemon* OR citrus OR cabbage* OR cauliflower* OR pepper* OR carrot* OR olive* OR onion* OR sago)) OR "sauerkraut" OR "table olive*" OR pickle* OR "kimchi" OR "paocai" OR torshi) **OR** TITLE-ABS-KEY (((Ferment* OR cultur*) W/6 (tea OR teas OR juice*)) OR "beer" OR "wine" OR cider* OR shochu OR kombucha* OR "pulque" OR puer OR "pu-er*" OR "fuzhuan" OR "dark tea*" OR "yellow tea*" OR coffee OR shalgam OR hardaliye) **OR** TITLE-ABS-KEY (((Ferment* OR cultur*) W/6 (soy OR soya OR bean* OR pea OR peas OR lentil* OR chickpea* OR legume* OR pulse* OR poi)) OR "soy* sauce*" OR "soybean paste*" OR miso* OR tempeh* OR tempe OR "natto" OR "doenjang" OR "doubanjiang" OR douchi OR "gochujang" OR cheonggukjang OR tsukemono OR garri) **OR** TITLE-ABS-KEY (((Ferment* OR cultur* OR leaven*) W/6 (cereal* OR grain* OR wheat* OR oat OR oats OR rice* OR millet* OR sorghum* OR maize* OR rye OR barley* OR chia OR oilseed* OR teff)) OR "bread" OR "sourdough" OR "crispbread" OR "boza" OR "ogi" OR dosa OR "tarhana" OR "buckwheat" OR "spelt" OR "einkorn" OR "quinoa" OR "amaranth" OR "tef" OR "bushera" OR chica OR chicha OR choujiu OR injera OR mahewu OR ogiri OR pozol OR ugba) **OR** TITLE-ABS-KEY (((Ferment* OR cultur*) W/6 (condiment* OR relish* OR horseradish OR dressing* OR seasoning* OR sauce* OR cocoa* OR tuber OR "acetic acid")) OR chocolate* OR vinegar* OR "tabasco" OR "sriracha" OR "Worcestershire" OR "Worcester")

#2 TITLE-ABS-KEY (((food OR *nutrient* OR eating OR nutrit*) W/6 (intake* OR habit* OR behavior* OR pattern* OR consumption OR suppl* OR ingestion)) OR diet* OR meal*)

#3 Functional strings specific to each PIMENTO WG3 review

#4 #1 AND #2 AND #3 *(add combination of string numbers to the field in advanced search in the field "Combined queries…")*

#5 TITLE-ABS-KEY (random* OR sham OR placebo*) OR TITLE-ABS-KEY ((singl* OR doubl*) W/1 (blind* OR dumm* OR mask*)) OR TITLE-ABS-KEY ((tripl* OR trebl*) W/1 (blind* OR dumm* OR mask*)) OR TITLE-ABS-KEY (control* W/3 (study OR studies OR trial* OR group*)) OR TITLE-ABS-KEY (clinical W/3 (study OR studies OR trial*)) OR TITLE-ABS-KEY (Nonrandom* OR "non random*" OR non-random* OR quasi-random* OR quasirandom*) OR TITLE-ABS-KEY (phase W/3 (study OR studies OR trial*)) OR TITLE-ABS-KEY ((crossover OR cross-over) W/3 (study OR studies OR trial*)) OR TITLE-ABS-KEY ((multicent* OR multi-cent*) W/3 (study OR studies OR trial*)) OR TITLE-ABS (allocated) OR TITLE-ABS-KEY (("open label" OR open-label) W/5 (study OR studies OR trial*)) OR TITLE-ABS-KEY ((equivalence OR superiority OR non-inferiority OR noninferiority) W/3 (study OR studies OR trial*)) OR TITLE-ABS-KEY ("pragmatic study" OR "pragmatic studies") OR TITLE-ABS-KEY ((pragmatic OR practical) W/3 trial*) OR TITLE-ABS-KEY ((quasiexperimental OR quasi-experimental) W/3 (study OR studies OR trial*)) OR TITLE (trial) OR KEY (trial)

#6 TITLE-ABS-KEY (observational W/3 (study OR studies OR design OR analysis OR analyses)) OR TITLE-ABS-KEY (cohort*) OR TITLE-ABS-KEY (prospective W/7 (study

OR studies OR design OR analysis OR analyses)) OR TITLE-ABS-KEY (("follow up" OR followup) W/7 (study OR studies OR design OR analysis OR analyses)) OR TITLE-ABS-KEY ((longitudinal OR longterm OR (long W/1 term)) W/7 (study OR studies OR design OR analysis OR analyses OR data)) OR TITLE-ABS-KEY (retrospective W/7 (study OR studies OR design OR analysis OR analyses OR data OR review)) OR TITLE-ABS-KEY ((case W/1 control) OR (case W/1 comparison) OR (case W/1 controlled)) OR TITLE-ABS-KEY (case-referent W/3 (study OR studies OR design OR analysis OR analyses)) OR TITLE-ABS-KEY (population W/3 (study OR studies OR analysis OR analyses)) OR TITLE-ABS-KEY (descriptive W/3 (study OR studies OR design OR analysis OR analyses)) OR TITLE-ABS-KEY ((multidimensional OR (multi W/1 dimensional)) W/3 (study OR studies OR design OR analysis OR analyses)) OR TITLE-ABS-KEY (cross W/1 sectional W/7 (study OR studies OR design OR research OR analysis OR analyses OR survey OR findings)) OR TITLE-ABS-KEY ((natural W/1 experiment) OR (natural W/1 experiments)) OR TITLE-ABS-KEY (quasi W/1 (experiment OR experiments OR experimental)) OR TITLE-ABS-KEY (("non experiment" OR nonexperiment OR "non experimental" OR nonexperimental) W/3 (study OR studies OR design OR analysis OR analyses)) OR TITLE-ABS-KEY (prevalence W/3 (study OR studies OR analysis OR analyses)) OR TITLE-ABS-KEY ("case series") OR TITLE-ABS-KEY (case W/3 (report OR reports OR study OR studies OR histories))

#7 TITLE-ABS-KEY ("systematic review")

#8 #5 OR #6 OR #7 *(add combination of string numbers to the field in advanced search in the field "Combined queries…")*

#9 #4 AND #8 *(add combination of string numbers to the field in advanced search in the field "Combined queries…")*

#10 (KEY (animal* OR nonhuman)) AND NOT (KEY (human*))

#11 #9 AND NOT #10 *(add combination of string numbers to the field in advanced search in the field "Combined queries…")*

#12 (KEY (infant* OR child*)) AND NOT (KEY (adult* OR aged))

#13 #11 AND NOT #12 *(add combination of string numbers to the field in advanced search in the field "Combined queries…")*

Limit #13 to English using the language filter

Limit #13 to 1970 - 2023 using the Year filter (Range from 1970 to 2023)

Search in Cochrane Library

#1* ((ferment* OR cultur* OR leaven*) NEAR/6 (food* OR drink* OR beverage* OR product*) OR (starter NEXT culture*)) OR (((ferment* OR culture* OR sour*) NEAR/6 (milk OR dairy OR cream*)) OR buttermilk OR cheese* OR yoghurt OR yogurt OR yoghourt OR yakult OR quark OR kefir OR lassi OR kumis OR koumiss OR kajmak OR airag OR ayran OR calpis OR borhani OR chal OR doogh OR kvass OR skyr OR amasi OR bouza OR butter* OR chal OR filmjolk OR kishk OR labne*) OR (((Ferment* OR cultur* OR cured) NEAR/6 (meat* OR fish* OR seafood* OR shellfish OR sausage*)) OR "salami" OR "pepperoni" OR peperoni OR "chorizo" OR "cervelat" OR "mettwurst" OR "summer sausage" OR "sucuk" OR (dried NEXT meat*) OR (dried NEXT sausage*) OR (dry NEXT sausage*) OR (fish NEXT sauce*) OR "shrimp paste" OR "shrimp sauce" OR "oyster sauce" OR "prosciutto" OR "pancetta" OR "saucisson" OR sucuk) OR (((Ferment* OR cultur*) NEAR/6 (fruit* OR vegetable* OR coconut* OR almond* OR hazelnut* OR nut OR cucumber* OR lemon* OR citrus OR cabbage* OR cauliflower* OR pepper* OR carrot* OR olive* OR onion* OR sago)) OR "sauerkraut" OR (table NEXT olive*) OR pickle* OR "kimchi" OR "paocai" OR torshi) OR (((Ferment* OR cultur*) NEAR/6 (tea OR teas OR juice*)) OR "beer" OR "wine" OR cider* OR shochu OR kombucha* OR "pulque" OR puer OR pu-er* OR "fuzhuan" OR (dark NEXT tea*) OR (yellow NEXT tea*) OR coffee OR shalgam OR hardaliye) OR (((Ferment* OR cultur*) NEAR/6 (soy OR soya OR bean* OR pea OR peas OR lentil* OR chickpea* OR legume* OR pulse* OR poi)) OR (soy* NEXT sauce*) OR (soybean NEXT paste*) OR miso* OR tempeh* OR tempe OR "natto" OR "doenjang" OR "doubanjiang" OR douchi OR "gochujang" OR cheonggukjang OR tsukemono OR garri) OR (((Ferment* OR cultur* OR leaven*) NEAR/6 (cereal* OR grain* OR wheat* OR oat OR oats OR rice* OR millet* OR sorghum* OR maize* OR rye OR barley* OR chia OR oilseed* OR teff)) OR "bread" OR "sourdough" OR "crispbread" OR "boza" OR “ogi” OR dosa OR "tarhana" OR "buckwheat" OR "spelt" OR "einkorn" OR "quinoa" OR "amaranth" OR "tef" OR "bushera" OR chica OR chicha OR choujiu OR injera OR mahewu OR ogiri OR pozol OR ugba) OR (((Ferment* OR cultur*) NEAR/6 (condiment* OR relish* OR horseradish OR dressing* OR seasoning* OR sauce* OR cocoa* OR tuber OR "acetic acid")) OR chocolate* OR vinegar* OR "tabasco" OR "sriracha" OR "Worcestershire" OR "Worcester")

#2* (((food OR macronutrient* OR eating) NEAR/6 (intake* OR habit* OR behavior* OR pattern*)) OR diet* OR intake OR ingestion OR suppl* OR consumption OR meal* OR nutrient* OR nutrit*)

#3* Functional strings specific to each PIMENTO WG3 review

#4* #1 AND #2 AND #3 in Trials and in Reviews

Choose in the field "Limits" the following options**: (a) Content type: "Cochrane Reviews" and "Trials"; (b) Date published on the Cochrane Library: Between "January 1970" and "August 2023"

*Search with filter "Title Abstract Keyword"
**Use results from both Cochrane Reviews and Trials for data selection
